# Diagnostic value of plasma p-tau181, NfL, and GFAP in a clinical setting cohort of prevalent neurodegenerative dementias

**DOI:** 10.1186/s13195-022-01093-6

**Published:** 2022-10-12

**Authors:** Simone Baiardi, Corinne Quadalti, Angela Mammana, Sofia Dellavalle, Corrado Zenesini, Luisa Sambati, Roberta Pantieri, Barbara Polischi, Luciano Romano, Matteo Suffritti, Giuseppe Mario Bentivenga, Vanda Randi, Michelangelo Stanzani-Maserati, Sabina Capellari, Piero Parchi

**Affiliations:** 1 Department of Experimental, Diagnostic and Specialty Medicine (DIMES) University of Bologna, Bologna, Italy; 2 IRCCS Istituto delle Scienze Neurologiche di Bologna, Via Altura 1/8, 40139 Bologna, Italy; 3 Department of Biomedical and Neuromotor Sciences University of Bologna (DIBINEM), Bologna, Italy; 4 Emilia-Romagna Regional Blood Bank, Immunohematology and Transfusion Medicine Service, Bologna Metropolitan Area, Bologna, Italy

**Keywords:** Frontotemporal dementia, FTLD, Alzheimer disease, Lewy bodies, Corticobasal syndrome, Progressive supranuclear palsy, RT-QuIC, Tau, Alpha-synuclein

## Abstract

**Background:**

Increasing evidence supports the use of plasma biomarkers of neurodegeneration and neuroinflammation to screen and diagnose patients with dementia. However, confirmatory studies are required to demonstrate their usefulness in the clinical setting.

**Methods:**

We evaluated plasma and cerebrospinal fluid (CSF) samples from consecutive patients with frontotemporal dementia (FTD) (*n* = 59), progressive supranuclear palsy (PSP) (*n* = 31), corticobasal syndrome (CBS) (*n* = 29), dementia with Lewy bodies (DLB) (*n* = 49), Alzheimer disease (AD) (*n* = 97), and suspected non-AD physiopathology (*n* = 51), as well as plasma samples from 60 healthy controls (HC). We measured neurofilament light chain (NfL), phospho-tau181 (p-tau181), and glial fibrillary acid protein (GFAP) using Simoa (all plasma biomarkers and CSF GFAP), CLEIA (CSF p-tau181), and ELISA (CSF NfL) assays. Additionally, we stratified patients according to the A/T/N classification scheme and the CSF α-synuclein real-time quaking-induced conversion assay (RT-QuIC) results.

**Results:**

We found good correlations between CSF and plasma biomarkers for NfL (rho = 0.668, *p* < 0.001) and p-tau181 (rho = 0.619, *p* < 0.001). Plasma NfL was significantly higher in disease groups than in HC and showed a greater increase in FTD than in AD [44.9 (28.1–68.6) vs. 21.9 (17.0–27.9) pg/ml, *p* < 0.001]. Conversely, plasma p-tau181 and GFAP levels were significantly higher in AD than in FTD [3.2 (2.4–4.3) vs. 1.1 (0.7–1.6) pg/ml, *p* < 0.001; 404.7 (279.7–503.0) vs. 198.2 (143.9–316.8) pg/ml, *p* < 0.001]. GFAP also allowed discriminating disease groups from HC. In the distinction between FTD and AD, plasma p-tau181 showed better accuracy (AUC 0.964) than NfL (AUC 0.791) and GFAP (AUC 0.818). In DLB and CBS, CSF amyloid positive (A+) subjects had higher plasma p-tau181 and GFAP levels than A− individuals. CSF RT-QuIC showed positive α-synuclein seeding activity in 96% DLB and 15% AD patients with no differences in plasma biomarker levels in those stratified by RT-QuIC result.

**Conclusions:**

In a single-center clinical cohort, we confirm the high diagnostic value of plasma p-tau181 for distinguishing FTD from AD and plasma NfL for discriminating degenerative dementias from HC. Plasma GFAP alone differentiates AD from FTD and neurodegenerative dementias from HC but with lower accuracy than p-tau181 and NfL. In CBS and DLB, plasma p-tau181 and GFAP levels are significantly influenced by beta-amyloid pathology.

**Supplementary Information:**

The online version contains supplementary material available at 10.1186/s13195-022-01093-6.

## Background

Recent technological advances, allowing for an ultrasensitive measure of molecules associated with neurodegeneration and neuroinflammation, have significantly contributed to the identification of non-invasive blood biomarkers for neurodegenerative dementias [1]. Although the emerging plasma biomarkers mainly correspond to those previously investigated in the cerebrospinal fluid (CSF), the association between biomarker levels in the two biofluids may vary [2].

Several studies demonstrated the high value of plasma phosphorylated tau at threonine 181, 217, and 231 (p-tau181, p-tau217, p-tau231) in distinguishing patients with autopsy-confirmed Alzheimer disease (AD) from negative controls [3–7], with a diagnostic accuracy close to the one provided by CSF analysis [3–5]. Moreover, plasma p-tau levels correlated with both tau- and Aβ-pathology burdens detected by positron emission tomography (PET) [8] or by CSF Aβ42 [9] in dementia with Lewy bodies (DLB), supporting the clinical use of plasma p-tau as a marker of AD co-pathology.

Similarly, neurofilament light chain (NfL), an unspecific marker of axonal injury, showed higher values in frontotemporal dementia (FTD) than in AD and DLB [10–12] and a strong association between the values in the CSF and blood [11]. In contrast, studies investigating plasma Aβ42/Aβ40 provided heterogeneous results [4, 13, 14], partly attributed to the different assays used for analyses [15], but overall indicating that the dosage of Aβ species in plasma is less accurate than the CSF analysis in distinguishing AD from other neurodegenerative dementias. Finally, glial fibrillary acid protein (GFAP), a marker of astrocytosis, has attracted recent attention because of preliminary evidence indicating a better performance of the plasma biomarker than the CSF counterpart in detecting AD pathology [16, 17], even in the preclinical or mild cognitive impairment (MCI)-AD stages [14, 17–20].

However, moving toward the clinical implementation of these blood biomarkers requires an in-depth evaluation of their diagnostic value, as an isolated marker or in combination, across the broad spectrum of neurodegenerative dementias. Moreover, given the frequent occurrence of mixed brain pathologies in dementia [21], the assessment of biomarker accuracy should account for the effect of overlapping comorbidities. Here, we performed a head-to-head comparison of the diagnostic performance of plasma p-tau181, NfL, and GFAP in AD, FTD, progressive supranuclear palsy (PSP), corticobasal syndrome (CBS), DLB, and a group of cognitively impaired individuals with suspected non-AD physiopathology (SNAP). We correlated plasma and CSF values and stratified each group for AD co-pathology according to the A/T/N classification and for Lewy body (LB) co-pathology using the α-synuclein (α-syn) real-time quaking-induced conversion (RT-QuIC) assay.

## Materials and methods

### Study design and patient classification

We retrospectively analyzed plasma and CSF samples from 316 consecutive FTD, PSP, CBS, DLB, AD, and SNAP patients and plasma samples from 60 healthy controls (HC) submitted to the Neuropathology Laboratory (NP-Lab) at the Institute of Neurological Science of Bologna, Italy, between 2005 and 2021 (2015–2020 for the AD group).

We reviewed the results of the diagnostic work-up for each patient, including clinical charts, neuropsychological testing, neuroimaging, and CSF AD core biomarkers. Neuropsychological evaluation was conducted as described [22]. According to the consensus criteria, clinical diagnoses were established by expert agreement [23–31]. In the FTD, PSP, CBS, DLB, and AD groups, only patients with a “probable” diagnosis according to the internationally established criteria were included in the study cohort to pursue the highest likelihood between the clinical diagnoses and the underlying pathologic processes. Cases lacking thorough clinical information (*n* = 59) and those without sufficient plasma or CSF for the analyses (*n* = 152) were excluded. No individuals with severe systemic illnesses were included in the study cohort.

Patients belonging to the frontotemporal lobar degeneration (FTLD) spectrum comprised the largest group (*n* = 119) and included participants with FTD (*n* = 59), PSP (*n* = 31), and CBS (*n* = 29). In the FTD group, we also distinguished between those with “pure” cognitive phenotypes (*n* = 43) from those with associated motor features (“plus” phenotypes, *n* = 16), namely amyotrophic lateral sclerosis (ALS) and parkinsonism. Pure FTD phenotypes included the following clinical syndromes: behavioral variant FTD (bvFTD, *n* = 33), non-fluent variant primary progressive aphasia (nfvPPA, *n* = 6), and logopenic variant PPA (lvPPA, *n* = 2), while two subjects presented with mixed bvFTD/PPA features. The FTD+parkinsonism (*n* = 8) and FTD+ALS (*n* = 8) groups included patients who met the criteria for bvFTD and/or PPA but also showed either extrapyramidal signs (in the presence of a mixed phenotype or not fully satisfying the criteria for CBS or PSP diagnosis) or upper and lower motor neuron impairment, respectively [22, 25]. Forty-nine patients were diagnosed as probable DLB/MCI-LB. All 97 individuals fulfilling the clinical criteria for AD/MCI-AD had a characteristic AD CSF biomarker profile supporting the clinical diagnosis (i.e., pathological values of Aβ42/40, p-tau/Aβ42 and t-tau/Aβ42 ratios according to in-house cutoffs) [32]. SNAP patients (*n* = 51) were cognitively impaired at neuropsychological testing, had normal CSF Aβ42/40 ratio, positive neurodegeneration biomarkers (as defined by neuroimaging and/or CSF findings), and did not fulfill the criteria for “probable” FTD, PSP, CSB, AD, and DLB. Finally, controls included a group of healthy (i.e., medical history not relevant for significant diseases/medications) blood donors (HC). Before blood collection, all HC underwent medical evaluation, including a standardized interview to exclude neurological symptoms.

### CSF collection, processing, and biomarker analyses

CSF samples were obtained by a lumbar puncture at the L3/L4 or L4/L5 intervertebral level and handled by experienced personnel at the NP-Lab. Samples showing signs of blood contamination (even minimal) were centrifuged at 2000*g* for 10 min at room temperature. Each sample (supernatant or non-centrifuged CSF) was divided into aliquots and stored in polypropylene tubes at − 80 °C until analysis.

#### AD core biomarker measurements

CSF t-tau, p-tau181, Aβ42, and Aβ40 were measured by automated chemiluminescent enzyme immunoassay on the Lumipulse G600II platform (Fujirebio, Gent, Belgium). The inter-assay coefficients of variation (CVs) were < 8% for all biomarkers. The Aβ42/40 ratio was calculated as described [33]. Pathological values for the AD core markers were determined according to validated cutoff values [32]. In particular, an Aβ42/40 ratio < 0.65, a p-tau/Aβ42 ratio > 0.08, and t-tau/Aβ42 ratio > 0.52 were considered supportive of AD.

#### Neurofilament light chain

NfL was quantified by a validated commercial enzyme-linked immunosorbent assay (NfL ELISA kit, IBL, Hamburg, Germany) [34]. The mean intra- and inter-assay CVs were 2% and 10%, respectively.

#### Glial fibrillary acid protein

GFAP was analyzed using the commercial SiMOA GFAP discovery kit on SiMOA SR-X platform. The mean intra- and inter-assay CVs were 9% and 12%.

#### α-Synuclein real-time quaking-induced conversion assay

To investigate the presence of α-syn seeding activity in the CSF, we performed the RT-QuIC assay according to our previously reported protocol [35, 36], with minor modifications. Briefly, we ran the same positive and negative controls throughout all experiments to limit the possible batch-to-batch variations of α-syn activity and the intrinsic plate-to-plate experimental variability. We normalized the relative fluorescent units for every time point for the maximum intensity reached by the positive control. Each CSF sample was run in quadruplicates and deemed positive when at least 2 out of 4 replicates reached the threshold. The latter corresponded to 30% of the median fluorescent peak values of the four positive control replicates included in each plate. The cutoff was set at 30 h. When only one replicate crossed the threshold, the analysis was considered “unclear” and repeated up to three times. The α-syn RT-QuIC assay results were previously reported for 37 out of 49 DLB patients [35].

### Plasma collection and biomarker analyses

For each participant, EDTA plasma samples were collected, aliquoted, and stored at − 80 °C according to standard procedures. All blood analyses were performed on a SiMOA SR-X analyzer platform (Quanterix, Billerica, MA, USA). Plasma NfL, p-tau181, and GFAP were measured with the SiMOA NF-light advantage, SiMOA p-tau181 advantage V2, and SiMOA GFAP discovery kits (i.e., the same used for GFAP quantification in CSF), respectively. The mean intra-assay and inter-assay CVs were respectively 5% and 15% for NfL, 9% and 18.5% for p-tau181, and 7% and 19% for GFAP. The samples were analyzed randomly to avoid bias due to the effect of inter-assay variability on specific patient groups.

### Genetic analysis

We screened all FTLD patients with a positive familial history of dementia defined by the presence of at least one dementia case among the first-degree relatives and/or those with a clinical history compatible with early-onset dementia (*n* = 57) for variants in 27 dementia-associated genes, including *GRN*, *MAPT*, *TARDBP*, and *FUS*, as previously reported [37]. In the same patient group, we also screened for the presence of the *C9orf72* repeat expansion using the 2-step strategy with southern blotting confirmation, as previously described [38].

*APOE* analysis was performed through PCR product digestion at 37 °C with the restriction enzyme HhaI (Thermo Fisher Scientific, Waltham, MA, USA) and visualized on 3.5% Metaphor agarose gel with GelStar nucleic acid gel stain or by targeted next-generation sequencing as previously reported [37].

### Statistical analysis

Statistical analyses were performed using the software Graphpad Prism 8.4 and Stata SE 14.2.

In the descriptive analysis, continuous variables were presented with mean and standard deviation (SD) or median and interquartile range (IQR) depending on the data distribution. The Shapiro-Wilk test was used to evaluate the normal data distribution. The categorical variables were presented as absolute (*n*) and relative frequency (%). The Student *t*-test, Mann-Whitney *U* test, one-way analysis of variance (followed by Bonferroni post hoc test), and Kruskal-Wallis test (followed by Dunn’s post hoc analysis) were used to compare the continuous variables between the groups. The chi-square test was used to compare categorical variables between the groups.

The biomarker levels were not normally distributed and were natural logarithm transformed, allowing linear model testing. Spearman’s correlation (rho) was used to evaluate the association between CSF and plasma values of each biomarker and clinical and genetic parameters with biomarker levels. Plasma and CSF biomarker values (dependent variables) were compared between the diagnostic groups (independent variable) with multivariate general linear models adjusting for age and sex. Receiver operating characteristic (ROC) analyses were performed, and sensitivity and specificity with relative 95% confidence intervals (95% CI) were calculated to evaluate the diagnostic accuracy of each biomarker in discriminating between the clinical groups. The optimal cutoff value for each biomarker was chosen using Youden’s Index. The De Long test was used to compare the areas under the curve between ROC curves. The differences were considered statistically significant at a *p*-value < 0.05, and all hypotheses were tested directionally at a 95% confidence level.

## Results

### Participant characteristics

The demographic characteristics of the study cohort and the results of genetic and CSF biomarker analyses are summarized in Tables 1 and 2 and Additional file 1: Table S1.

**Table 1:** Demographic characteristics of the study cohort

	Number	Female, *n* (%)	Age at CSF/plasma collection, years	Time from onset to CSF/plasma collection, months
FTD^#^	59	34 (57.6)^a,b^	62.9 (8.9)^c–f^	34.3 (33.5)^a,e^
PSP	31	11 (35.5)^g^	69.2 (10.2)^h^	51.5 (33.1)^i^
CBS	29	18 (62.1)^b^	71.3 (7.2)^h^	43.2 (37.4)
DLB	49	14 (28.6)^f,j^	73.7 (6.7)^h,i,k^	65.3 (53.9)^f,i^
AD	97	54 (55.7)	67.8 (9.3)^h^	41.7 (34.9)^i^
SNAP	51	25 (49.0)	66.2 (9.5)	26.6 (25.1)
HC	60	26 (43.3)	61.7 (4.9)	–

Continuous variables are expressed as mean (SD)

*FTD* frontotemporal dementia, *PSP* progressive supranuclear palsy, *CBS* corticobasal syndrome, *DLB* dementia with Lewy bodies, *AD* Alzheimer disease, *SNAP* suspected non-AD physiopathology, *HC* healthy controls

^a^vs. PSP ≤ 0.05

^b^vs. DLB ≤ 0.01

^c^vs. PSP ≤ 0.01

^d^vs. CBS ≤ 0.001

^e^vs. DLB ≤ 0.001

^f^vs. AD ≤ 0.01

^g^vs. CBS ≤ 0.05

^h^vs. HC ≤ 0.001

^i^vs. SNAP ≤ 0.001

^j^vs. SNAP ≤ 0.05

^k^AD ≤ 0.001

^#^Included cases with FTD + ALS and FTD + parkinsonism

**Table 2:** Clinical and genetic features across diagnostic groups

Diagnostic groups	FTD^#^	PSP	CBS	DLB	AD	SNAP
*N*	59	31	29	49	97	51
Onset < 65 years, %	40 (67.8)^a–e^	13 (41.9)	11 (37.9)	14 (28.6)^d^	47 (48.4)	19 (37.3)
MMSE score, /30	24.8 (3.9)^d^	25.4 (5.1)^d^	24.3 (5.5)	23.1 (5.3)	21.9 (6.2)^e^	25.6 (3.7)
Brief Mental Deterioration Battery	0.1 (1.5)	0.5 (1.1)	0.3 (1.3)	− 0.2 (1.2)	− 0.5 (1.4)	0.1 (1.6)
CDR score ≥ 1, %^°^	40 (81.6)^e,f^	17 (54.8)^d^	19 (67.9)	34 (69.4)	66 (77.6)^g^	29 (56.8)
CDR score ≥ 2, %^°^	18 (41.9)	6 (19.4)	5 (17.9)	14 (28.6)	33 (38.8)	12 (23.5)
ADL/IADL impairment, %	51 (86.4)^f,h,i^	18 (58.1)^j^	24 (82.7)^g^	34 (69.4)	74 (76.2)^e^	28 (54.9)
CSF A+, %	2 (3.4)^c,k,l^	4 (12.9)^b,g,l,m^	14 (48.3)^i,l^	18 (36.7)^i,l^	97 (100)^i^	0 (0.0)
CSF T+, %	2 (3.4)^k,l,m^	3 (9.7)^b,l^	12 (41.4)^e,l,m^	7 (14.3)^l^	94 (96.9)^i^	2 (3.9)
CSF N+, %	6 (10.2)^l^	1 (3.2)^l,n^	7 (24.1)^l^	7 (14.3)^l^	73 (75.2)^i^	6 (11.7)
Positive α-syn RT-QuIC test, %	0 (0.0)^c,l^	0 (0.0)^c,d^	1 (3.4)^c^	47 (95.9)^i,l^	15 (15.5)^i^	1 (1.9)
APOE ε4, positive/tested, %	12/51 (23.5)^g^	6/31 (19.3)	6/27 (22.2)	13/49 (26.5)^e^	35/94 (37.2)^i^	5/50 (10.0)
Monogenic disease, positive/tested, %	18/37 (48.6)*	0/8 (0.0)	0/12 (0.0)	–	–	–

Continuous variables are expressed as mean (SD)

*FTD* frontotemporal dementia, *PSP* progressive supranuclear palsy, *CBS* corticobasal syndrome, *DLB* dementia with Lewy bodies, *AD* Alzheimer disease, *SNAP* suspected non-AD physiopathology

^a^vs. PSP ≤ 0.05

^b^vs. CBS ≤ 0.01

^c^vs. DLB ≤ 0.001

^d^vs. AD ≤ 0.05

^e^vs. SNAP ≤ 0.01

^f^vs. PSP ≤ 0.001

^g^vs. SNAP ≤ 0.05

^h^vs. vs. DLB ≤ 0.05

^i^vs. SNAP ≤ 0.001

^j^vs. CBS ≤ 0.05

^k^vs. CBS ≤ 0.001

^l^vs. AD ≤ 0.001

^m^vs. DLB ≤ 0.05

^n^vs. CBS ≤ 0.05

^°^CDR score was available only in 49 out of 59 FTD, 28 of 29 CBS, and 85 of 97 AD patients

^*^C9orf72 (*n* = 7), *GRN* (*n* = 6), *FUS* (*n* = 2), *TARDBP* (*n* = 1), *OPTN* (*n* = 1), *LRP10* (*n* = 1). ^#^Included cases with FTD + ALS and FTD + parkinsonism

FTD and HC were significantly younger at biosample collection than the other diagnostic groups, but for SNAP (*p* ≤ 0.01 for all comparisons). There were no significant differences in the mean age between the AD, CBS, PSP, and DLB groups, except for DLB patients being older than AD and SNAP patients (*p* < 0.001). The time between disease onset and biosample collection differed between DLB and FTD (*p* < 0.001), PSP and FTD (*p* = 0.02), DLB and AD (*p* = 0.006), SNAP and PSP or DLB (*p* < 0.001 for both comparisons), and SNAP and AD groups (*p* = 0.03). Females were underrepresented in the DLB (vs. FTD, *p* = 0.002; vs. CBS, *p* = 0.004, vs. AD, *p* = 0.002, vs. SNAP *p* = 0.04) and PSP (vs. FTD, *p* = 0.046; vs. CBS, *p* = 0.039) groups.

In the FTD group, 18 patients had a monogenic disease linked to the most prevalent mutations in the *GRN* (*n* = 6) and *C9orf72* (*n* = 7) genes. There was a slightly higher prevalence of genetic cases in the FTD “plus” than in the “pure” phenotype (37.5% vs. 27.9%), but the difference did not reach statistical significance. As expected, the AD group showed the highest prevalence of *APOE* ε4 carriers (Table 2). The mean MMSE scores were lower in AD than in FTD (*p* = 0.026), PSP (*p* = 0.028), and SNAP (*p* = 0.006) patients. We found no difference in the mean clinical dementia rating (CDR) between the groups except for a trend toward higher scores in AD patients than in those with PSP (*p* = 0.065) and SNAP (*p* = 0.105).

### CSF and plasma NfL, GFAP, and p-tau181 in the study cohort

In plasma, age was associated with NfL levels in the controls (rho = 0.608, *p* < 0.001), with p-tau181 values in AD (rho = − 0.313, *p* = 0.002), and with GFAP concentrations in FTD (rho = 0.366, *p* = 0.005) and PSP (rho = 0.596, *p* < 0.001) groups. Sex showed no effect on blood and CSF biomarker values.

**Fig. 1:**
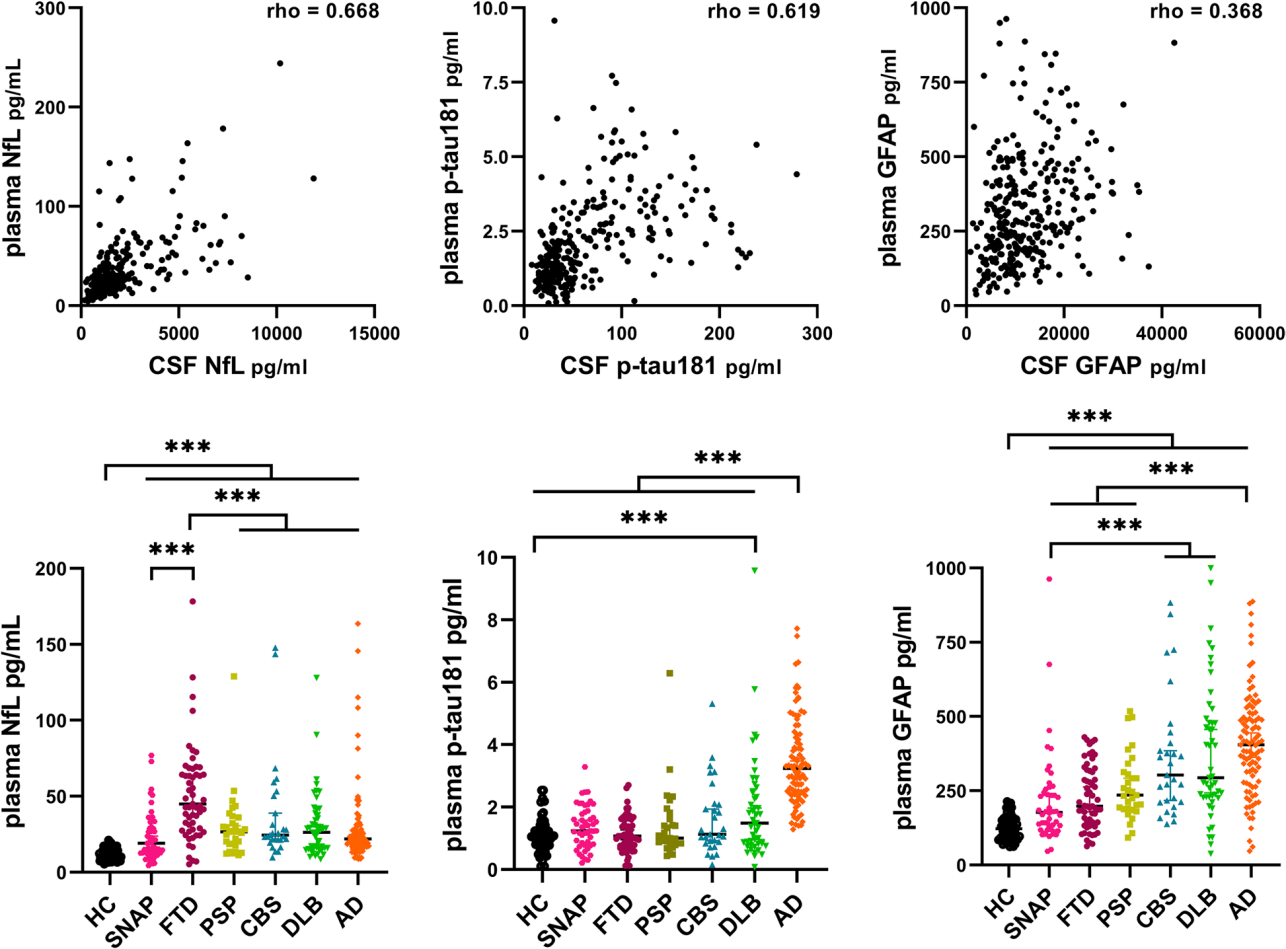
Correlations between plasma and CSF values of each biomarker, and plasma NfL, p-tau181, and GFAP levels across diagnostic groups and healthy controls. ***p ≤ 0.001

In line with previous studies, we found good correlations between CSF and plasma NfL values overall (rho = 0.668, *p* < 0.001) (Fig. 1). In particular, there was a strong association in FTD (rho = 0.749, *p* < 0.001), SNAP (rho = 0.722, *p* < 0.001), and DLB (rho = 0.720, *p* < 0.001) and a moderate correlation in CBS (rho = 0.635, *p* < 0.001).

In CSF, NfL levels were significantly increased in FTD compared to all other groups (vs. PSP, DLB, AD, and SNAP, *p* < 0.001; vs. CBS, *p* = 0.01) (Additional file 1: Table S2). We found similar trends in plasma, with significantly higher values in FTD compared to all other diagnostic groups (*p* < 0.001) (Fig. 1, Additional file 1: Table S3). HC showed the lowest plasma NfL levels, resulting in significant differences with each diagnostic group (*p* < 0.001). In the SNAP group, we detected higher plasma NfL levels in individuals presenting with prominent behavioral changes compared to those with “pure” cognitive impairment [26.7 (12.7–39.8) vs. 15.7 (9.9–22.9) pg/ml, *p* = 0.12] (Additional file 1: Fig. S1).

Plasma and CSF p-tau181 showed a good overall correlation in the study cohort (rho = 0.619, *p* < 0.001).

As expected, we found the highest CSF p-tau181 levels in the AD group (*p* < 0.001 for all comparisons). Additionally, CBS patients showed higher biomarker levels than those with FTD, PSP and SNAP (*p* < 0.001 for all comparisons), and DLB (*p* = 0.006). In line with CSF findings, plasma p-tau181 levels were significantly higher in AD than in the other groups (*p* < 0.001 for all comparisons) and in DLB compared with FTD (*p* = 0.002). HC had significantly lower plasma p-tau181 levels than AD and DLB (*p* < 0.001 for both comparisons) patients but comparable to those in the FTD, PSP, CBS, and SNAP groups.

Among the biomarkers analyzed, GFAP showed the weakest correlation between plasma and CSF levels (rho = 0.325, *p* < 0.001). Accordingly, we found only a weak to fair correlation in the FTD (rho = 0.299, *p* = 0.021), SNAP (rho = 0.368, *p* = 0.010), and DLB (rho = 0.452, *p* = 0.001) participants. In CSF, GFAP did not differ among the diagnostic groups. Still, plasma levels were significantly higher in AD than in FTD, PSP, and SNAP (*p* < 0.001 for all comparisons) (Fig. 1). Finally, HC had the lowest plasma GFAP values compared with the other groups (*p* < 0.001 for all comparisons).

### CSF and plasma biomarkers in the FTD phenotypic spectrum

After stratifying FTD patients according to the phenotype, there were no significant differences in either CSF or plasma biomarker values (Additional file 1: Table S4). Although both plasma and CSF NfL showed the highest levels in the FTD+ALS, they did not reach statistical significance, likely because of the few cases analyzed and the variability within the group itself.

A monogenic disease was most represented in the FTD group (Table 2); therefore, we also compared pathogenic mutations in the former group, besides evaluating biomarker levels between genetic and sporadic cases. We found higher CSF NfL values in the genetic cohort than in the sporadic group (*p* = 0.013) and, within genetic FTD, a higher increase in plasma GFAP levels in *GRN* mutation carriers than in individuals with *C9orf72* or other mutations (*p* = 0.029 and *p* = 0.036, respectively) (Additional file 1: Table S5).

### Diagnostic accuracy of plasma NfL, p-tau181, and GFAP

In the discrimination between the HC and disease groups, ROC curve analysis demonstrated high accuracy for both plasma NfL and GFAP, with an area under the curve (AUC) values ranging from 0.948 (vs. CBS) to 0.778 (vs. SNAP) for NfL and from 0.942 (vs. CBS) to 0.740 (vs. SNAP) for GFAP (Additional file 1: Fig. S2). Plasma p-tau181, instead, showed the overall highest accuracy in distinguishing HC from AD (AUC 0.971), but had a lower performance than NfL and GFAP in separating HC from the other groups (AUC range 0.533–0.661) (Table 3).

**Table 3:** CSF and plasma biomarkers’ sensitivity, specificity, and accuracy in distinguishing the major diagnostic categories

	Analyte	Biosample	Cutoff (pg/ml)	Sens. (%) (95% CI)	Spec. (%) (95% CI)	AUC (95% CI)
HC vs. disease groups	NfL	Plasma	> 16.6	76.9 (72.0–81.2)	90.0 (79.9–95.3)	0.897 (0.864–0.930)
	GFAP	Plasma	> 163.1	81.2 (76.5–85.1)	85.0 (73.9–91.9)	0.880 (0.846–0.915)
	p-tau181	Plasma	> 1.57	54.0 (48.5–59.5)	86.7 (75.8–93.1)	0.716 (0.658–0.774)
FTD vs. other diseases^a^	NfL	CSF	> 1801	71.2 (58.6–81.2)	78.6 (72.5–83.7)	0.784 (0.710–0.857)
		Plasma	> 31.3	72.9 (60.4–82.6)	74.3 (67.9–79.8)	0.761 (0.686–0.836)
AD vs. other diseases^b^	p-tau181	CSF	> 65.5	91.8 (84.6–95.8)	90.5 (85.1–94.1)	0.954 (0.931–0.978)
		Plasma	>1.98	86.6 (78.4–92.0)	80.0 (73.3–85.4)	0.889 (0.851–0.928)
	GFAP	CSF	> 7958	83.5 (74.9–89.6)	34.5 (27.8–41.9)	0.584 (0.514–0.653)
		Plasma	> 313.6	73.2 (63.6–81.0)	64.9 (57.4–71.7)	0.703 (0.638–0.768)

^a^PSP+CBS+DLB+AD

^b^PSP+CBS+DLB+FTD

**Fig. 2:**
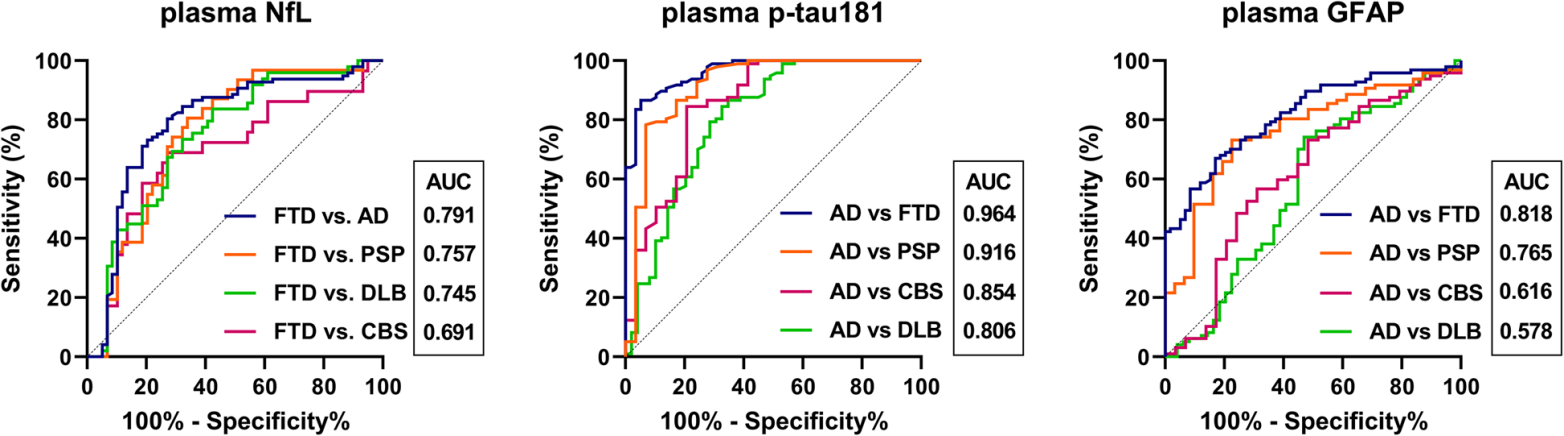
Diagnostic accuracy of plasma NfL, p-tau181, and GFAP

In the distinction between the disease groups, plasma NfL showed a moderate accuracy in differentiating FTD from the other disease groups (AD+PSP+CBS+DLB) (cutoff > 31.3 pg/ml, sensitivity 72.9%, specificity 74.3%, AUC 0.761) with a similar diagnostic performance against each group (Fig. 2). As for the distinction from HC, plasma p-tau181 showed the highest accuracy in discriminating AD from the other disease groups (FTD+PSP+CBS+DLB: cutoff > 1.98 pg/ml, sensitivity 86.6%, specificity 80.0%, AUC 0.889), in particular from FTD (AUC 0.964) and PSP (AUC 0.916), while its diagnostic value was lower for CBS (AUC 0.854) and DLB (AUC 0.806).

Although less accurate than p-tau181, plasma GFAP also distinguished AD from the other disease groups (AUC 0.889 vs. 0.703, *p* < 0.001), but more efficiently against FTD (AUC 0.818) and PSP (AUC 0.765) than against CBS (AUC 0.616) and DLB (AUC 0.578).

The comparison of biomarker performance between CSF and plasma revealed an almost identical accuracy for NfL in the discrimination between FTD and AD, PSP, or CBS, with CSF NfL performing slightly better only in the distinction between FTD and DLB (AUC 0.831 vs. 0.756, *p* = 0.020) (Additional file 1: Fig. S3). According to the ROC curves, CSF p-tau181 showed a greater accuracy than plasma p-tau181 only in the distinction between AD and DLB (AUC 0.951 vs. 0.806, *p* < 0.001). In contrast to p-tau181, GFAP showed higher overall diagnostic accuracy in plasma than in CSF (AUC 0.703 vs. 0.584, *p* = 0.005) (Table 3), especially in the distinction between AD and FTD (AUC 0.632 vs. 0.818, *p* < 0.001) and between AD and PSP (AUC 0.575 vs. 0.765, *p* = 0.008).

### AD core biomarkers

Patients in the AD group showed the highest CSF t-tau and p-tau181 values and the lowest mean Aβ42/40 ratio (Additional file 1: Table S2). CSF Aβ40 was positively associated with age (rho = 0.187, *p* < 0.001). Analyzing the whole disease cohort, we found a significant negative correlation between Aβ42/40 ratio and plasma p-tau181 (rho − 0.631, *p* < 0.001, rho − 0.197, *p* = 0.017 after excluding the T+ cases) and between Aβ42/40 ratio and plasma GFAP (rho − 0.471, *p* < 0.001, rho − 0.189, *p* = 0.022 after excluding the T+ cases).

As previously demonstrated, the diagnostic accuracy of both plasma p-tau181 and GFAP was lower in distinguishing AD from CBS and DLB than FTD and PSP. Notably, the former groups had greater prevalence of amyloid co-pathology as disclosed by the A/T/N classification (A+: CBS vs. FTD, *p* < 0.001; vs. PSP, *p* = 0.004; DLB vs. FTD, *p* < 0.001; vs. PSP, *p* = 0.023) (Table 2). Therefore, we evaluated plasma biomarkers in CBS and DLB groups after stratifying individuals according to their amyloid status (A+ vs. A−) (Table 4, Additional file 1: Fig. S4). In both groups, plasma p-tau181 and GFAP showed significantly higher levels in A+ cases. In contrast, there was no association between plasma NfL and amyloid status. We then repeated the ROC analyses for p-tau181 and GFAP after excluding the patients with CSF A+ and found a significant improvement in the diagnostic accuracy for AD vs. CBS (p-tau181 AUC from 0.854 to 0.972; GFAP AUC from 0.616 to 0.758) and AD vs. DLB (p-tau181 AUC from 0.806 to 0.905; GFAP AUC from 0.578 to 0.707).

**Table 4:** Summary of baseline characteristics and plasma biomarkers in dementia with Lewy bodies and corticobasal syndrome groups according to CSF amyloid (A+/–) status

	DLB			CBS		
	A+ (*n* = 17)	A− (*n* = 31)	*p* value	A+ (*n* = 14)	A− (*n* = 15)	*p* value
Age, years	73.4 (6.3)	73.6 (7.1)	0.937	71.4 (8.0)	71.3 (6.6)	0.973
Sex (female)	7 (38.9%)	7 (22.6%)	0.326	7 (50.0%)	11 (73.3%)	0.263
MMSE, /30	19.2 (6.3)	25.1 (3.5)	< 0.001	23.6 (6.8)	25.1 (4.1)	0.829
NfL (pg/mL)	29.3 (17.1–29.3)	20.4 (14.6–35.5)	0.129	24.0 (16.4–53.1)	27.9 (21.6–53.3)	0.477
p-tau181 (pg/mL)	2.5 (1.9–3.5)	1 (0.7–1.8)	< 0.001	2.0 (1.0–3.2)	1.0 (0.7–1.3)	0.004
GFAP (pg/mL)	485.1 (380.9–705.3)	242.7 (211.1–417.2)	< 0.001	380.7 (325.3–642.9)	225.5 (178.8–302.9)	0.007

Age is expressed as mean (SD), while biomarker data are presented as median (IQR)

### Prevalence of CSF α-syn seeding activity in the diagnostic groups

The RT-QuIC revealed a positive α-syn seeding activity in the CSF of 47 out of 49 (95.9%) patients in the DLB group, in 15/97 (15.5%) in AD, and a single subject also showing a CSF AD profile (A+T+N+) in the CBS group. Additionally, we found α-syn seeding activity in the CSF of a SNAP patient presenting with major (amnestic multiple domain) cognitive decline and visual hallucinations, fulfilling the criteria for “possible” DLB. In contrast, the α-syn RT-QuIC assay was invariably negative in FTD and PSP subjects.

Within the DLB group, most cases showed a full (4/4) response (*n* = 41, 87.2%), 4 a 3/4 positivity (8.5%), and 2 a 2/4 response (4.2%). We found a significantly lower percentage of 4/4 (*n* = 5, 33.3%, *p* < 0.001) response and a higher prevalence of 3/4 (*n* = 6, 40%, *p* = 0.009) and 2/4 (*n* = 4, 26.7%, *p* = 0.026) responses in AD patients compared to those with DLB.

After stratifying the AD subgroup according to α-syn co-pathology, we found no significant difference in demographic features or plasma biomarker levels (Table 5).

**Table 5:** Summary of baseline characteristics and plasma biomarker levels of the Alzheimer disease group according to LB α-syn status

	α-syn+ (*n* = 15)	α-syn− (*n* = 82)	*p* value
Age, years	67.7 (8.5)	67.9 (9.5)	0.939
Sex (female)	6 (40.0%)	48 (58.5%)	0.259
MMSE, /30	22.1 (6.9)	21.9 (6.1)	0.695
Plasma NfL (pg/mL)	20.1 (15.5–27.7)	22.0 (17.1–27.9)	0.550
Plasma p-tau181 (pg/mL)	3.0 (2.4–5.0)	3.3 (2.4–4.2)	0.807
Plasma GFAP (pg/mL)	338.9 (245.1–475.8)	413.6 (306.1–509.7)	0.228

Age and MMSE are expressed as mean (SD), while biomarker data are presented as median (IQR range)

### Association between plasma biomarkers, clinical variables, and APOE status

In the overall disease cohort, all plasma biomarkers were associated with CDR score (*p* < 0.001) and impairment of daily life activities (*p* < 0.001). Additionally, we found that both p-tau181 and GFAP values were negatively associated with MMSE score (rho = − 0.269, *p* < 0.001, and rho = − 0.294, *p* < 0.001, respectively). The BBDM score was correlated only with GFAP (rho = − 0.194, *p =* 0.008), while p-tau181 was associated with the *APOE ε4* status (*p* = 0.001). In the SNAP group, plasma NfL levels were higher in individuals with impairment of daily life activities (*p* = 0.054) and correlated with the MMSE (rho = − 0.327, *p* = 0.028) and CDR scores (rho = 0.304, *p* = 0.016). Of note, after stratifying the DLB group according to the amyloid status, we found a lower mean MMSE score in the DLB A+ group than in DLB A− (Table 4), indicating a possible contribution of AD co-pathology worsening cognitive performance.

## Discussion

The present study confirms and expands previous evidence on the diagnostic value of plasma NfL, p-tau181, and GFAP, in a clinical cohort representative of the whole spectrum of prevalent neurodegenerative dementias. We investigated the association between plasma and CSF levels for each biomarker. Moreover, we measured CSF AD core biomarker and α-syn seeding activity by RT-QuIC analyses to evaluate the impact on plasma biomarkers of AD co-pathology in the FTLD and DLB subgroups and of LB pathology in AD. In line with the results of previous studies [3, 11], we found a significant association between plasma and CSF levels for most biomarkers. Plasma NfL levels were significantly higher in the clinical groups than in controls, especially in patients with FTD, resulting in a “fair” discriminative ability with the other clinical groups (AUC ranging from 0.791 to 0.691).

In contrast, plasma NfL did not accurately distinguish between PSP, CBS, AD, and DLB. These findings support the current view that plasma NfL could be an effective biomarker for screening individuals manifesting neuropsychiatric symptoms to distinguish neurodegenerative from non-neurodegenerative disorders [39, 40]. They also confirm the overall low to moderate discriminatory power of plasma NfL in the distinction between FTD and its mimics, especially at disease onset (i.e., AD and DLB) [41].

We found higher NfL levels in the CSF, but not in the plasma, of FTLD individuals carrying pathogenic mutations than in sporadic cases, which could depend on the frequent association with motor neuron disease. However, given the relatively low number of patients analyzed and the lack of a definitive explanation for the discrepancy between the results obtained in CSF and plasma, additional studies should investigate the consistency of this finding in larger cohorts.

Both plasma p-tau181 and GFAP reached the highest levels in AD. However, these biomarkers showed a different ability to separate disease groups from controls and a divergent diagnostic accuracy when tested in CSF and plasma. Notably, we found increased plasma p-tau181 levels in CSF A+ individuals in all clinical groups, not only in the AD group. In contrast, there were no differences between the CSF A− group and controls, providing evidence of biomarker specificity for AD pathology and supporting the idea that p-tau181 might be a valuable marker of AD co-pathology. The latter finding is particularly relevant for DLB because of the high prevalence of mixed DLB+AD pathology documented by neuropathology in large cohorts [42–44]. Similarly, CBS is notoriously associated with several distinct histopathologies that are difficult to predict on clinical features [45, 46]. Of note, we found increased plasma p-tau181 in CSF T+ individuals with DLB or CBS and, to a lesser extent, also in those A+/T−, confirming the initial evidence that plasma p-tau181 levels strongly correlate with the Aβ burden [3, 47] and already increase in the early disease stage [3]. Despite the good correlation in the whole cohort, plasma p-tau181 values showed a highly variable correlation with CSF levels, depending on the diagnostic group. This result likely depends on the restrictive selection criteria we applied to the AD group, which included almost only patients with CSF T+. It partly justifies the better diagnostic performance of p-tau181 in CSF than in plasma, discriminating the AD group from FTD, DLB, and PSP. In our cohort, plasma and CSF p-tau181 showed a similar accuracy for AD vs. CBS, probably because the latter group also included a relatively high percentage of CSF T+ cases.

Unlike p-tau181, a marker of AD pathology, GFAP is not associated with a specific neuropathologic process. Accordingly, we found higher plasma GFAP values in all disease groups (although to a variable extent) than in controls, allowing us to separate patients from controls with a good to excellent accuracy (0.740–0.942).

Previous studies consistently documented a more significant increase in GFAP in plasma, serum, and whole blood in AD than in FTD [14, 16, 48]. In contrast, there is no agreement on whether and to what extent the biomarker is increased in FTD compared to controls [17, 49, 50], probably due to the contribution of multiple clinical and genetic factors. Our FTD cohort included both *GRN* carriers (10% of cases) and patients with severe disease (7% with CRD-FTLD ≥ 3), two conditions known to be associated with increased biomarker levels [49, 51].

As expected [14, 17, 52–55], individuals of the AD group showed the most consistent increase in plasma GFAP levels, allowing their discrimination from the other disease group with an overall moderate accuracy (AUC 0.578–0.818), although lower than that shown by p-tau181. The modest correlation between CSF and plasma GFAP values in our cohort is consistent with data reported in previous studies [17, 18].

Overall, the results of the present study suggest that plasma p-tau181 is more suitable than NfL and GFAP in the differential diagnosis of AD from disorders belonging to the FTLD spectrum and DLB, irrespective of the disease severity. In contrast, plasma NfL and GFAP are adequate, either as single biomarkers or in combination, to distinguish neurodegenerative dementias from healthy individuals. These conclusions have largely confirmatory value but demonstrate their adaptability to the clinical setting where the diagnosis is uncertain, often because of the contribution of mixed dementias/pathologies. Additionally, our data support the usefulness of plasma p-tau181 and GFAP in screening for AD co-pathology, even in cases with alternative primary dementias.

In the present study, we also confirmed the high clinical value of the CSF α-syn RT-QuIC assay in a broad spectrum of neurodegenerative dementias, as a diagnostic tool for DLB and a marker of LB co-pathology in other diagnostic groups, particularly in AD. In the latter, the lack of significant effect of α-syn status on demographic and clinical features as well as on plasma biomarker levels could depend on the small sample size of our study population. Therefore it needs to be investigated further in larger AD cohorts.

Strengths of the study are the head-to-head comparison of multiple diseases that are representative of most neurodegenerative dementias evaluated in a single referral center, including a SNAP group with an uncertain diagnosis, the evaluation of various biomarkers in both CSF and plasma, and the analysis of the contribution of AD and LB co-pathologies to the biomarker results. In particular, we assessed α-syn seeding activity in CSF samples of the entire disease cohort by the RT-QuIC, an assay that demonstrated high diagnostic value for detecting Lewy body disease even in the prodromal stage [32, 36].

The present study has limitations: firstly, the sample size is heterogeneous across diagnostic groups, but it reflects the reality of consecutive dementia/neurodegenerative individuals submitted to a single laboratory. Secondly, AD pathology was determined by the CSF A/T/N profile and not by PET imaging which could have provided quantitative information about the burden of cerebral pathology (Aβ, tau). Thirdly, the control group lacks CSF and neuroimaging studies defining the A/T/N status. Lastly, due to the limited size of disease groups, patients were not stratified according to disease severity.

## Conclusions

Our study confirms, in the clinical setting, the high diagnostic value of plasma p-tau181 for distinguishing FTD from AD and that of NfL for discriminating between neurodegenerative dementias and HC, suggesting their combined use for diagnostic screening. Plasma GFAP alone has the combined value of distinguishing AD from FTD and disease groups from HC, but with lower accuracy than p-tau181 and NfL, respectively. Finally, clinicians should be aware that in non-AD groups, especially those in which co-existence of AD pathological change is a common finding, such as CBS and DLB, amyloid co-pathology significantly influences plasma p-tau181 and GFAP levels.

## Supplementary Information

**Additional file 1: Table S1.** Clinical features and results of indicative biomarkers in the DLB group. **Table S2.** CSF NfL, GFAP, and AD core biomarker values across diagnostic groups. **Table S3.** Plasma NfL, GFAP, and p-tau181 values across diagnostic groups. **Table S4.** CSF and plasma NfL, GFAP, and p-tau181 across FTD phenotypic continuum. **Table S5.** CSF and plasma NfL, GFAP, and p-tau181 in genetic and sporadic FTD. **Fig. S1.** Plasma NfL levels in the SNAP group according to the clinical presentation. **Fig. S2.** Accuracy of plasma NfL, p-tau181, and GFAP in discriminating between healthy controls and disease groups. **Fig. S3.** CSF and plasma diagnostic accuracies for each biomarker across disease groups. **Fig. S4.** Plasma biomarker levels in CBS and DLB according to the amyloid status (left) and ROC curves after excluding A+ cases in non-AD disease groups (right).

## Data Availability

The datasets used and analyzed during the current study are available from the corresponding author upon reasonable request.

## Abbreviations

α-syn: α-Synuclein
AD: Alzheimer disease
ALS: Amyotrophic lateral sclerosis
AUC: Area under the curve
BvFTD: Behavioral variant FTD
CBS: Corticobasal syndrome
CDR: Clinical dementia rating
CI: Confidence interval
CSF: Cerebrospinal fluid
CVs: Coefficients of variation
DLB: Dementia with Lewy body
FTD: Frontotemporal dementia
FTLD: Frontotemporal lobar degeneration
GFAP: Glial fibrillary acid protein
HC: Healthy controls
IQR: Interquartile range
LB: Lewy body
lvPPA: Logopenic variant PPA
MCI: Mild cognitive impairment
MMSE: Mini-Mental State Examination
NfL: Neurofilament light chain
nfvPPA: Non-fluent variant primary progressive aphasia
NP-Lab: Neuropathology Laboratory
PET: Positron emission tomography
PSP: Progressive supranuclear palsy
p-tau: Phosphorylated tau
ROC: Receiver operating characteristic
RT-QuIC: Real-time quaking-induced conversion
SD: Standard deviation
SNAP: Suspected non-AD physiopathology
