# Supplementary material for: Diagnostic value of plasma p-tau181, NfL, and GFAP in a clinical setting cohort of prevalent neurodegenerative dementias
Source: Alzheimers Res Ther. 2022 Oct 12;14:153. doi: 10.1186/s13195-022-01093-6 (PMC9555092; doi:10.1186/s13195-022-01093-6)

ADDITIONAL FILE 1

SUPPLEMENTARY MATERIALS

List of contents

Table S1…………………………………………………………………………………… 2

Table S2…………………………………………………………………………………… 3

Table S3…………………………………………………………………………………… 4

Table S4…………………………………………………………………………………… 5

Table S5…………………………………………………………………………………… 6

Fig. S1……………………………………………………………………………………… 7

Fig. S2……………………………………………………………………………………… 8

Fig. S3……………………………………………………………………………………… 9

Fig. S4……………………………………………………………………………………… 10

Table S1. Clinical features and results of indicative biomarkers in the DLB group.

Variable	N=49
Clinical core feature, No. (%)	
Fluctuations	17 (34.7)
Visual hallucinations	29 (59.2)
REM sleep behavior disorder	23 (46.9)
Parkinsonism	45 (91.8)
1 out 4 clinical core features	9 (18.4)
2 out 4 clinical core features	21 (42.9)
3 out 4 clinical core features	13 (26.5)
4 of 4 clinical core features	6 (12.2)
Biomarker, No. of patients with positive finding/No. tested (%)	
DaTscan	25/29 (86.2)
^{123}I-MIBG uptake at myocardial scintigraphy	10/11 (90.9)
REM sleep without atonia at PSG	10/14 (71.4)

Table S2. CSF NfL, GFAP and AD core biomarker values across diagnostic groups.

	FTD	PSP	CBS	DLB	AD	SNAP
n	59	31	29	49	97	51
NfL	2459 (1492-4676)[a-e]	1664 (1109-2086)[f]	1454 (1087-2481)[c]	1045 (789-1334)	1061 (863-1508)	1114 (676-2710)
GFAP	9118 (5613-14385)	10383 (8248-15379)	12817 (8269-18309)	10635 (6507-16309)	11917 (8515-17388)	9462 (5517-14541)
t-tau	269 (201-381)[d]	200 (154-284)[d]	309 (215-478)[d]	246 (170-251)[d]	640 (492-986)[e]	256 (182-353)
p-tau181	28 (23-36)[d,g]	30 (22-46)[d,g]	45 (31-81)[d-f]	31 (25-48)[d]	109 (82-153)[e]	31 (25-42)
Aβ42/40	0.93 (0.82-0.99)[c,d]	0.86 (0.78-0.97)[d,f]	0.71 (0.56-0.94)[d,e]	0.84 (0.56-0.93)[d,e]	0.43 (0.39-0.49)[e]	0.94 (0.87-1.00)
Aβ42	671 (533-671)[d]	585 (463-1016)[d]	578 (479-832)	607 (350-759)[h]	476 (369-592)[e]	778 (541-1000)
Aβ40	7510 (5732-9344)[d]	7731 (5657-11387)[d]	9191 (7159-11207)[j]	7348 (6679-9506)[d]	11165 (8891-13670)[e]	8567 (6450-10729)

Biomarker values are expressed in pg/ml as median (IQR range). [a] vs. PSP ≤0.001; [b] vs. CBS ≤0.01; [c] vs. DLB ≤0.001; [d] vs. AD ≤0.001; [e]NfL FTD vs SNAP < 0.001; [f] vs. DLB ≤0.05; [g] vs. CBS ≤0.001; [h] vs. SNAP ≤0.01; [j] vs. AD ≤0.05.

Table S3. Plasma NfL, GFAP and p-tau181 values across diagnostic groups.

	FTD	PSP	CBS	DLB	AD	SNAP	HC
n	59	31	29	49	97	51	60
NfL	44.9 (28.1-68.6)[a-f]	26.6 (18.5-36.0)[f]	24.5 (21.4-51.8)[f,g]	26.3 (15.9-38.2)[f]	21.9 (17.0-27.9)[f]	19.1 (12.6-28.7)[f]	10.1 (8.5-14.6)
GFAP	198.2 (143.9-316.8)[d,f]	235.2 (187.5-312.7)[d,f]	302.9 (211.2-428.2)[e,f]	293.6 (227.8-485.9)[e,f]	404.7 (279.7-503.0)[e,f]	177.4 (118.8-260.5)[f]	121.9 (91.2-156.3)
p-tau181	1.1 (0.7-1.6)[d]	1.0 (0.8-1.7)[d]	1.1 (0.9-2.1)[d]	1.5 (0.8-2.6)[d,f]	3.2 (2.4-4.3)[e,f]	1.2 (0.8-1.9)	1.0 (0.8-1.3)

Biomarker values are expressed in pg/ml as median (IQR range). [a] vs. PSP ≤0.001; [b] vs. CBS ≤0.001; [c] vs. DLB ≤0.001; [d] vs. AD ≤0.001; [e] vs. SNAP ≤0.001; [f] vs. HC ≤0.001; [g] vs. SNAP ≤0.01.

Table S4. CSF and plasma NfL, GFAP and p-tau181 across FTD phenotypic continuum.

	pure FTD				FTD *plus*		
phenotype	All	BvFTD	nfvPPA	lvPPA	All	FTD+park	FTD+ALS
n	43*	33	6	2	16	8	8
CSF							
NfL	2376 (1485-4405)	2150 (1389-4253)	4008 (2643-4872)	2190 (1920-2459)	3214 (1511-7063)	2776 (1660-6982)	4121 (1409-14846)
p-tau	28.0 (23.0-36.0)	27.0 (22.0-35.0)	33.5 (22.8-43.8)	38.0 (28.0-48.0)	29.0 (23.3-38.3)	26.0 (21.5-38.3)	31.0 (24.8-46.5)
GFAP	9351 (5594-16399)	8231 (5280-16052)	9759 (6350-15878)	-	5658 (4801-11577)	5939 (4862-7281)	5658 (3958-12180)
Plasma							
NfL	44.9 (26.7-64.3)	42.6 (26.5-66.2)	54.8 (25.2-67.3)	26.8 (16.6-37.0)	49.9 (34.3-113.1)	59.8 (43.0-96.9)	41.2 (29.2-292.6)
p-tau	1.0 (0.7-1.5)	1.0 (0.7-1.4)	0.9 (0.6-1.6)	0.9 (0.8-1.1)	1.3 (0.6-1.7)	1.2 (0.7-1.6)	1.3 (0.6-1.9)
GFAP	189.5 (130.9-273.0)	169.5 (134.5-282.4)	158.2 (120.8-219.3)	144.0 (102.1-185.9)	295.9 (180.5-337.5)	319.3 (263.5-365.8)	192.3 (160.0-326.5)

Biomarker values are expressed in pg/ml as median (IQR range). *Includes 2 cases with mixed BvFTD/PPA features.

Table S5. CSF and plasma NfL, GFAP and p-tau181 in genetic and sporadic FTD.

	genetic[#]				sporadic[#]
mutation	*C9orf72*	*GRN*	Others	All	
n	7	6	5	18	41
CSF					
NfL	4498 (1492-4844)	5604 (3910-7394)	3565 (1490-8153)	4502 (2085-6409)[a]	2150 (1390-3800)
p-tau	42 (16-51)	25 (20-28)	35 (28-42)	28 (22-43)	28 (23-34)
GFAP	9759 (5432-12180)	14121 (9332-31687)	8231 (5539-17150)	10690 (7346-16948)	6865 (5096-12723)
Plasma					
NfL	42.8 (28.1-69.1)	72.6 (63.9-79.4)	39.6 (17.1-84.8)	57.2 (30.5-76.1)	43.6 (26.9-64.3)
p-tau	1.4 (0.6-1.7)	0.9 (0.6-1.2)	0.7 (0.3-2.4)	0.9 (0.6-1.7)	1.1 (0.8-1.6)
GFAP	153.8 (132.6-187.7)[b]	284.9 (216.5-384.6)[c]	129.5 (91.4-240.7)	182.6 (130.9-273.0)	199.0 (150.4-324.3)

Biomarker values are expressed in pg/ml as median (IQR range). [a] vs. sporadic ≤0.05; [b] vs. genetic *GRN* ≤0.05; [c] vs. genetic Others ≤0.05. [#]The FTD-ALS phenotype was slightly more frequent in the genetic (22.2%) than sporadic group (9.7%).

Fig. S1. Plasma NfL levels in the SNAP group according with the clinical presentation.

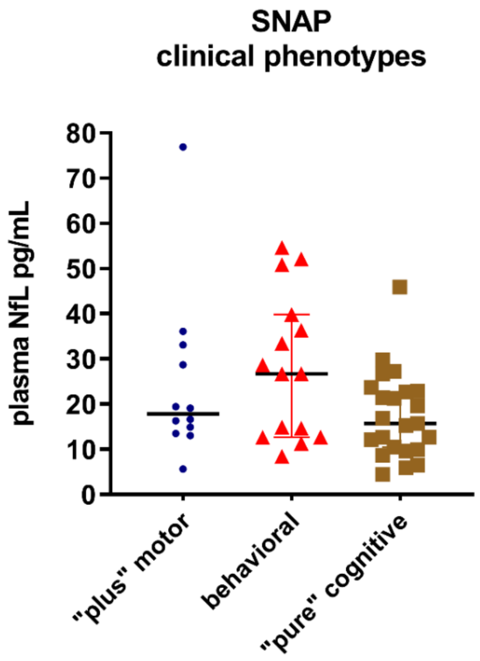

Fig. S2. Accuracy of plasma NfL, p-tau181 and GFAP in discriminating between healthy controls and disease groups.

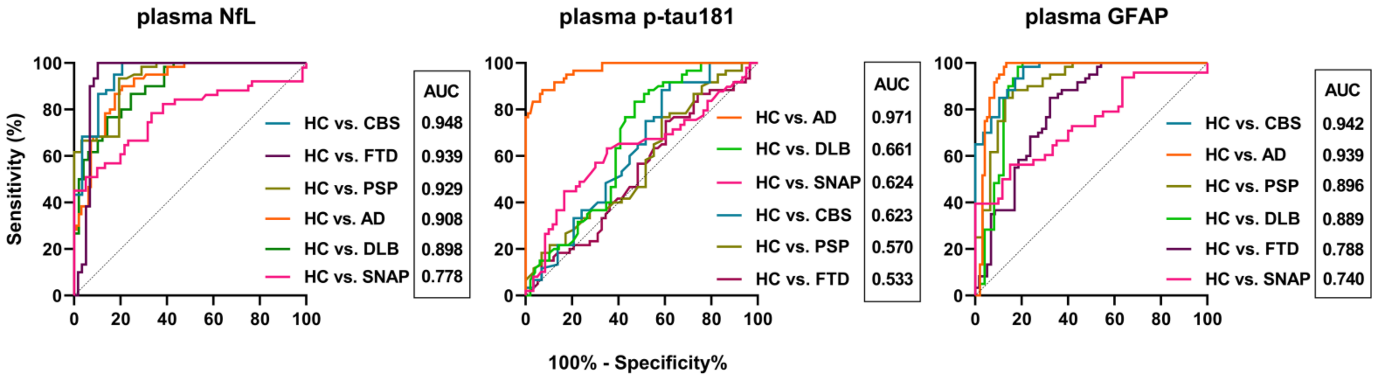

Fig. S3. Comparisons between CSF and plasma accuracy for each biomarker across disease groups.

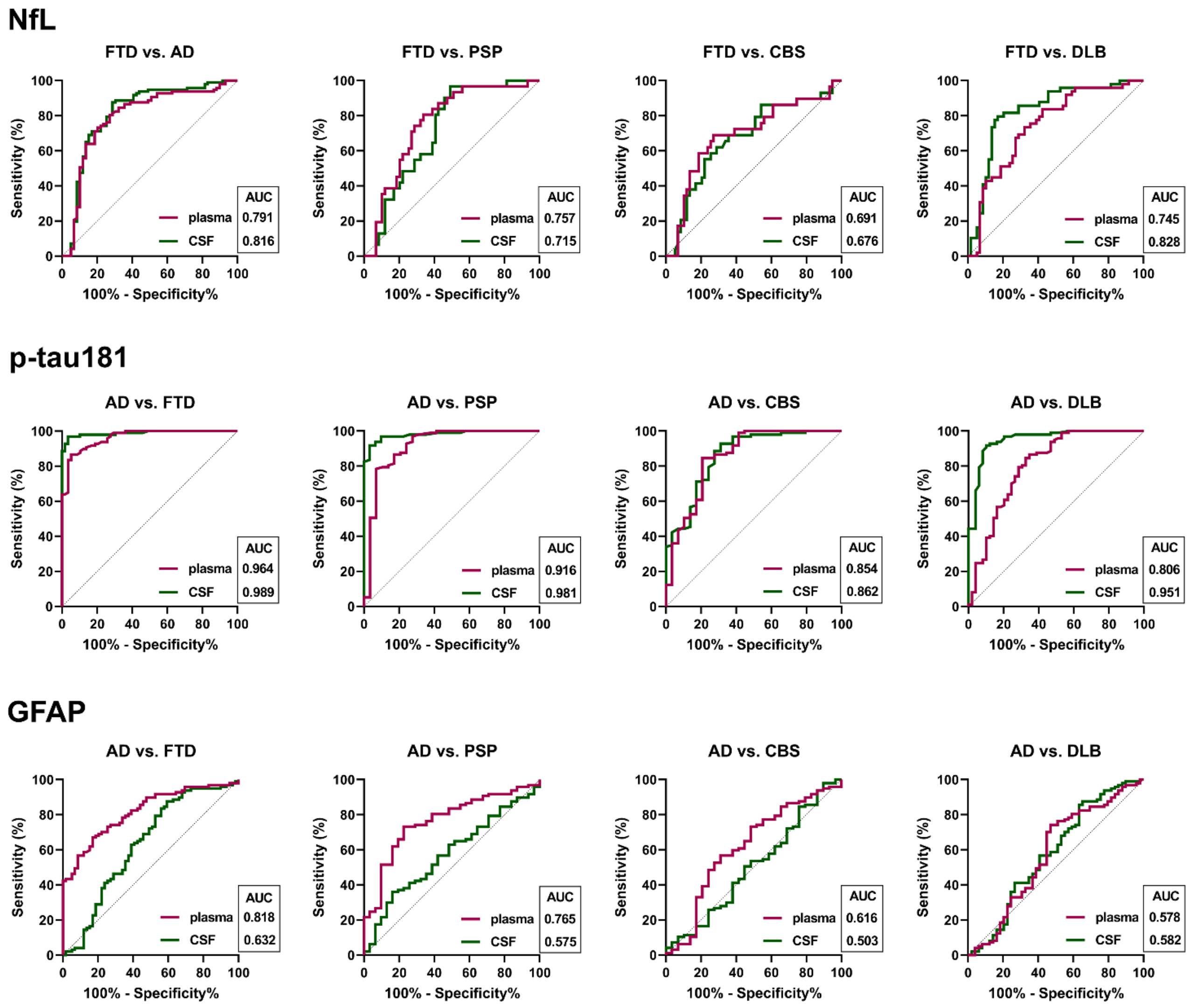

Fig. S4. Plasma biomarker levels in CBS and DLB according with the amyloid status (left) and ROC curves after excluding A+ cases in non-AD disease groups (right).

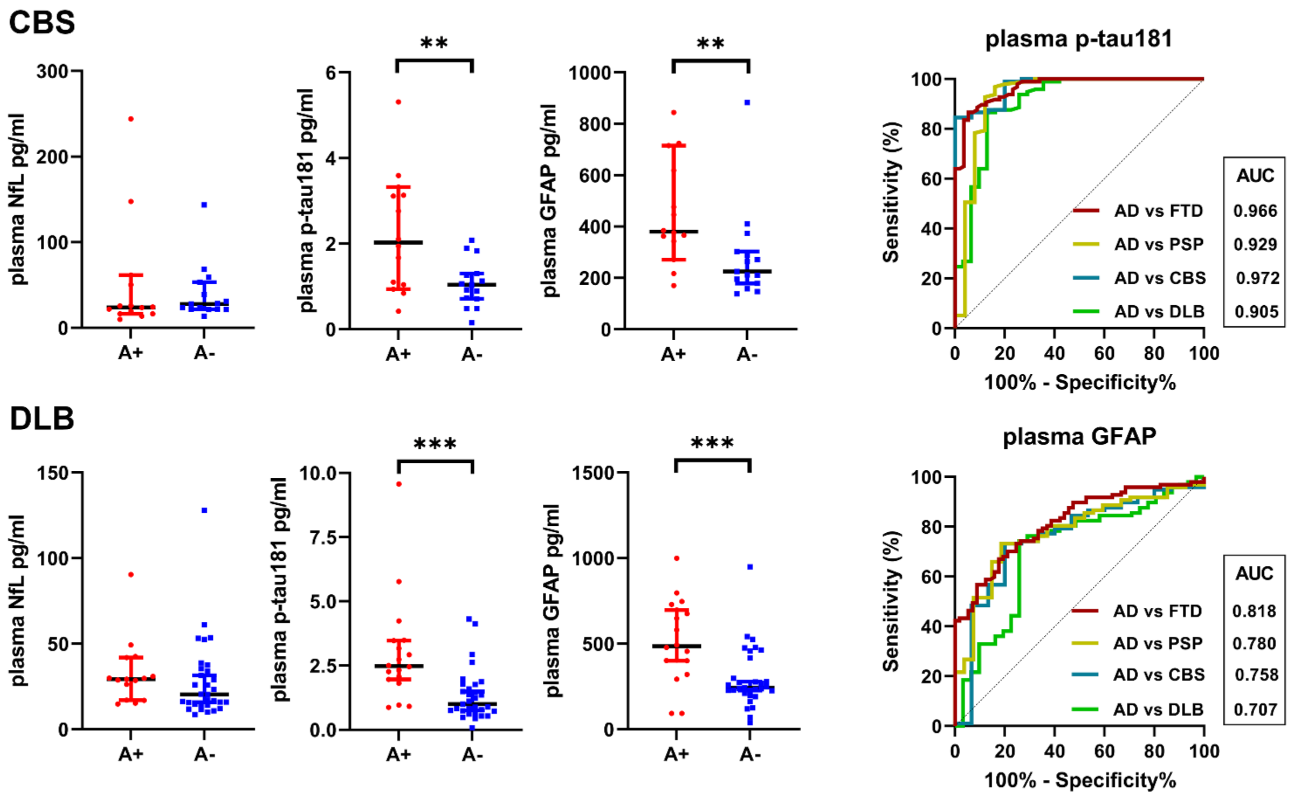

Supplement: Supplementary file 1 — Additional file 1: Table S1. Clinical features and results of indicative biomarkers in the DLB group. Table S2. CSF NfL, GFAP, and AD core biomarker values across diagnostic groups. Table S3. Plasma NfL, GFAP, and p-tau181 values across diagnostic groups. Table S4. CSF and plasma NfL, GFAP, and p-tau181 across FTD phenotypic continuum. Table S5. CSF and plasma NfL, GFAP, and p-tau181 in genetic and sporadic FTD. Fig. S1. Plasma NfL levels in the SNAP group according to the clinical presentation. Fig. S2. Accuracy of plasma NfL, p-tau181, and GFAP in discriminating between healthy controls and disease groups. Fig. S3. CSF and plasma diagnostic accuracies for each biomarker across disease groups. Fig. S4. Plasma biomarker levels in CBS and DLB according to the amyloid status (left) and ROC curves after excluding A+ cases in non-AD disease groups (right). [file 13195_2022_1093_MOESM1_ESM.pdf]
